# Short-Term Effect of Prosthesis Transforming Sensory Modalities on Walking in Stroke Patients with Hemiparesis

**DOI:** 10.1155/2016/6809879

**Published:** 2016-07-31

**Authors:** Dai Owaki, Yusuke Sekiguchi, Keita Honda, Akio Ishiguro, Shin-ichi Izumi

**Affiliations:** ^1^Research Institute of Electrical Communication, Tohoku University, 2-2-1 Katahira, Aoba-ku, Sendai 980-8577, Japan; ^2^Department of Physical Medicine and Rehabilitation, Tohoku University Graduate School of Medicine, 2-1 Seiryo-machi, Sendai 980-8575, Japan; ^3^Graduate School of Biomedical Engineering, Tohoku University, 2-1 Seiryo-machi, Sendai 980-8575, Japan

## Abstract

Sensory impairments caused by neurological or physical disorders hamper kinesthesia, making rehabilitation difficult. In order to overcome this problem, we proposed and developed a novel biofeedback prosthesis called* Auditory Foot* for transforming sensory modalities, in which the sensor prosthesis transforms plantar sensations to auditory feedback signals. This study investigated the short-term effect of the auditory feedback prosthesis on walking in stroke patients with hemiparesis. To evaluate the effect, we compared four conditions of auditory feedback from plantar sensors at the heel and fifth metatarsal. We found significant differences in the maximum hip extension angle and ankle plantar flexor moment on the affected side during the stance phase, between conditions with and without auditory feedback signals. These results indicate that our sensory prosthesis could enhance walking performance in stroke patients with hemiparesis, resulting in effective short-term rehabilitation.

## 1. Introduction

Rehabilitation includes physical therapy that enables long-term improvements through short-term efforts during daily interventions; it promotes mobility, improves functional ability, and improves the quality of life. During physical therapy, kinesthesia, that is, motion perception that “*I am moving*,” which is generated through the interaction dynamics between motor and sensory systems, that is, the “motor-sensory loop,” plays a crucial role in long-term motor learning as well as short-term motion generation. Thus, achievement of kinesthesia is essential for the rehabilitation of physical impairments and disabilities. However, sensory impairments caused by neurological or physical disorders hamper kinesthesia, making rehabilitation difficult.

**Figure 1:**
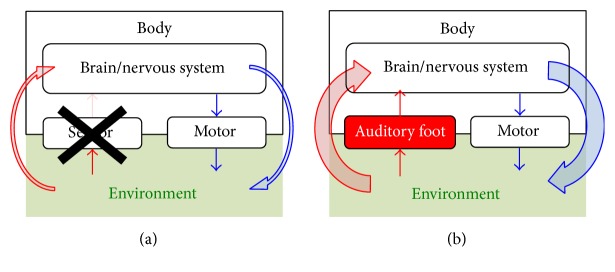
Schematic of the proposed rehabilitation for sensory impairments: (a) from the viewpoint of system engineering, sensory impairments are considered as* input failure* in a system, leading to dysfunction of the entire system. (b) The dysfunction can be corrected through* repair* or* replacement* by another input component, where enhancement of kinesthetic feedback or replacement with another sensory modality allows intervention for the dysfunction through reestablishment of motor-sensory loop.

For the rehabilitation of sensory impairments, we proposed a novel biofeedback prosthesis [1] that transforms weak or deficient kinesthetic feedback into an alternative sensory modality. From the viewpoint of system engineering, sensory impairments in humans are considered as* input failure* in a system, leading to dysfunction of the entire system; the dysfunction can be corrected through* repair* or* replacement* by another input component (Figure 1). In this situation, enhancement of kinesthetic feedback or replacement with another sensory modality allows intervention for the dysfunction and enables reestablishment of motor-sensory loop in patients undergoing rehabilitation.

Thus, the aim of this study was to verify the short-term effect of the prosthesis in transforming sensory modalities for patients with sensory impairments. In particular, we focused on an auditory feedback prosthesis that transformed plantar sensation in walking rehabilitation [1] for the following four reasons: plantar sensation, that is, the trajectory of the center of pressure (COP) on the plantar region and the magnitude of load, is an essential kinesthesia in walking [2–7]; in stroke patients with hemiparesis, the range of COP trajectories during walking is narrowed on the affected foot through the change of gait [8]; the time required for the cognitive resolution of auditory signals in the human brain (approximately 1 ms) is shorter than that required for the resolution of visual feedback signals (approximately 50–100 ms); and visual feedback systems, for example, a display showing visual feedback signals, constrains the posture of subjects, resulting in limited rehabilitation spaces and approaches.

**Figure 2:**
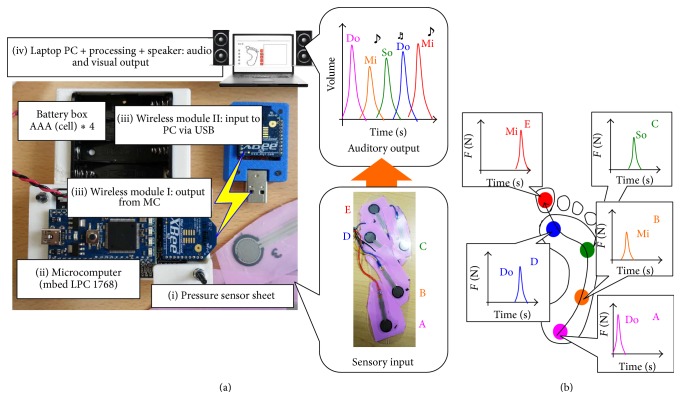
Overview of the proposed prosthesis transforming sensory modalities, called* Auditory Foot* [1]: (a) entire system of the sensor prosthesis, which consists of four components: (i) a pressure sensor sheet (input), (ii) a microcomputer (data processing), (iii) wireless communication devices (data transport from module I to module II), and (iv) a PC (output). (b) Protocol for transformation from pressure to auditory signals. The position of pressure sensors corresponds to “musical interval” (cf. Do, Mi, So, Do, and Mi) and the magnitude of pressure sensor values corresponds to “audio volumes.”

Previous studies have proposed auditory feedback systems for walking rehabilitation; Miyake [10] proposed the* Walk-Mate* system that utilizes the “mutual entrainment” of the timing of footsteps of a subject and an agent modeled on a computer system and showed that patients' as well as healthy subjects' gait were restored to a stable and natural walking state. Schauer and Mauritz [11] verified the timing effect of auditory signals at touchdown during walking rehabilitation for stroke patients. However, no previous studies focused on transforming the spatiotemporal pattern of loading on a foot to auditory feedback signals. Here, we applied our prosthesis, called* Auditory Foot* [1], which transforms multipoint cutaneous plantar sensations to auditory feedback signals (Figure 2), to walking rehabilitation in stroke patients with hemiparesis, and demonstrated the short-term effect of the prosthesis on the intervention.

## 2. Materials and Methods

### 2.1. Prosthesis Transforming Sensory Modalities:* Auditory Foot*

We proposed a sensor prosthesis called* Auditory Foot* [1] for transforming sensory modalities for waking rehabilitation. Auditory Foot transforms cutaneous plantar sensations to auditory feedback signals during walking. The entire system consists of four components (Figure 2(a)): (i) a pressure sensor sheet (input component), (ii) a microcomputer (data processing component), (iii) wireless communication devices (data transport component), and (iv) a PC (output component). The pressure sensor sheet consists of many pressure sensors (e.g., five sensors; Figure 2 middle bottom, Interlink Electronics: FSR402). The sheet structure allowed us to easily modify the distribution of sensor points for individuals and easily attach the sheet to physical or prosthetic feet or insert it into shoes. A microcomputer (mbed NXP LPC1768) converted analog data from pressure sensors to digital data and sent them to a wireless communication device (Xbee, Digital International: ZB RF module) via serial communication. Using Xbee, digital data from the microcomputer was transported to a laptop PC via wireless communication. In the laptop PC, the Processing software [12] computes digital data from the Xbee device and transformed them to auditory and visual outputs with a speaker and PC monitor, respectively.

In the Processing software, we designed a transformation protocol from plantar sensation to auditory signal outputs as follows: the position of pressure sensors corresponded to a musical interval (cf. Do, Mi, So, Do, and Mi) and the magnitude of pressure sensor values corresponded to audio volumes. Thus, auditory signals corresponded to the spatiotemporal pattern of loading on a foot, as shown in Figure 2(b).

### 2.2. Patients

We recruited stroke patients (6 men and 1 woman) with hemiparesis. Average age, height, and weight were 55 ± 12 years old, 168 ± 8 cm, and 67 ± 7 kg, respectively. Inclusion criteria were as follows: ability to walk without walking prosthetics, ability to understand instructions by physical therapists, and a first-time stroke due to either ischemic or hemorrhagic causes. Subjects with hemiplegia were excluded if they had any of the following: brainstem or cerebellar lesions, abnormal circulatory and respiratory status, abnormal mental status, or higher brain dysfunction. The Brunnstrom stage and Stroke Impairment Assessment Set (SIAS) [13] of the patients are shown in Table 1. Patients gave written informed consent before participating in the study, which was approved by the local ethics committee of Tohoku University Hospital, Japan.

### 2.3. Gait Assessment

**Figure 3:**
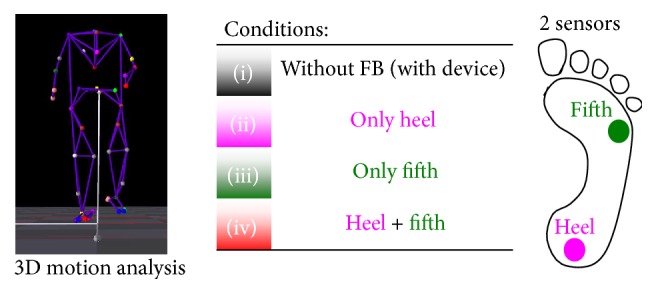
Measurement conditions: we compared four conditions with the use of two pressure sensors at the heel and fifth metatarsal (right): (i) without auditory feedback but with prosthesis, called “without” condition; (ii) auditory feedback from only the heel sensor, called “heel” condition; (iii) auditory feedback from only the fifth metatarsal sensor, called “fifth” condition; and (iv) auditory feedback from both the heel and fifth metatarsal sensors, called “all” condition. These four conditions were conducted in a random order.

For assessment of the effect of our prosthesis on gait in patients, we compared four conditions with the use of two pressure sensors at the heel and fifth metatarsal (Figure 3): (i) without auditory feedback but with prosthesis, called “without” condition; (ii) auditory feedback from only the heel sensor, called “heel” condition; (iii) auditory feedback from only the fifth metatarsal sensor, called “fifth” condition; and (iv) auditory feedback from both the heel and fifth metatarsal sensors, called “all” condition. Assessment was conducted in these four conditions in a random order. All patients were tested in each auditory feedback condition on the same day.

We asked participants to walk for 7 m and two to eight trials were conducted for each condition, including more than three walking periods (from touchdown of the affected foot to the next touchdown). To investigate unconscious effects, we did not instruct patients, for example, “apply load on sounding places.” In all measurements, the coordinates of each reflective marker were measured by the MAC 3D System (120 Hz) (Motion Analysis Corporation, Santa Rosa, CA, USA). Forty-one reflective markers were placed using adhesive tape on 12 segments according to the anatomical positions (Table 2). Ground reaction force (GRF) data were acquired at a 1200 Hz sampling rate using four 90 cm × 60 cm force plates (Anima Corporation, Chofu-shi, Tokyo, Japan). The three-dimensional coordinates and GRF data were smoothed using a bidirectional forth-order Butterworth low-pass filter with cut-off frequencies of 20 and 200 Hz, respectively. A twelve-segment model based on the anthropometric data suggested by Dumas et al. [14] was comprised of the feet, shanks, thighs, pelvis, thorax, upper arms, and forearms. The kinematic data at each joint in the lower extremities were calculated using the joint coordinate system [15]. Moreover, lower extremity joint kinetics was estimated using inverse dynamics [16]. The representative parameter for gait in patients with hemiparesis was extracted from kinematic and kinetic data, referring to the previous study by Kinsella and Moran [17]. The parameters were calculated with custom software in MATLAB (The MathWorks Incorporated, Natick, MA).

### 2.4. Dynamic Joint Stiffness

In this study, we used dynamic joint stiffness measurement [9], which has been proposed for the assessment of the* resistance* of a joint during human walking. The dynamic joint stiffness of a joint *k*_*i*_ is defined as the ratio of the variation of its moment Δ*τ*_*i*_ to the variation of the corresponding joint angle Δ*θ*_*i*_, described by the following equation:

(1) $$\begin{array}{c} k_{i}=\frac{\Delta \tau_{i}}{\Delta \theta_{i}}. \end{array}$$

We choose dynamic joint stiffness at the ankle joint as this measure can systematically evaluate the contribution of ankle function to walking gait when using our prosthesis. Evaluation of joint stiffness to demonstrate gait recovery has been reported in previous studies [18, 19]. An increased period of stiffness at the end of the stance phase implied effective pushing off the ground, and a decreased period of stiffness at the beginning of the stance phase implied shock absorption during gait.

### 2.5. Statistical Analysis

Repeated measures ANOVAs followed by Dunnett post hoc tests were used to reveal differences in gait parameters between the without (i) and the with auditory feedback conditions (ii)–(iv). A *p *value of 0.05 was set as the criterion for statistical significance. Statistical analyses were performed using a statistical software package (SPSS Ver.22, SPSS Inc., Chicago, IL, USA).

## 3. Results

### 3.1. Effect on Gait-Related Parameters

**Figure 4:**
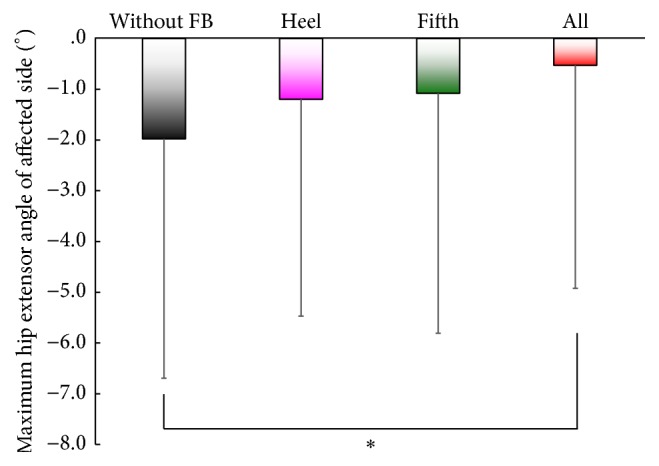
Effects on the maximum hip extension angle of the affected side during the stance phase. ^⁎^ *p* < 0.05, two-tailed. A significant difference was observed between (i) “without” and (iv) “all” conditions.

**Figure 5:**
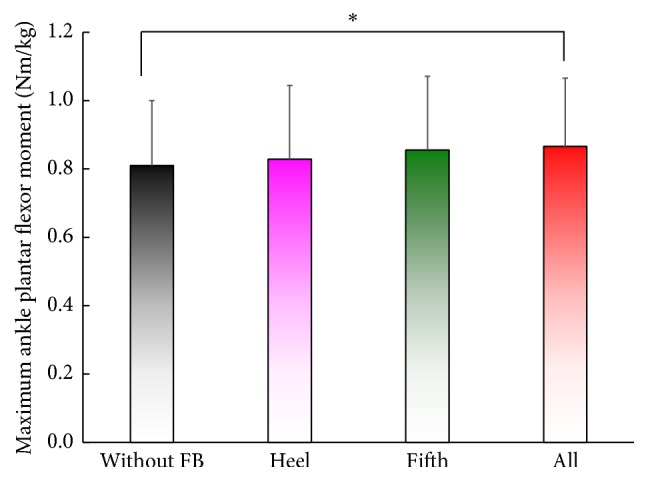
Effects on the maximum ankle plantar flexor moment of the affected side. ^⁎^ *p* < 0.05, two-tailed. A significant difference is found between (i) “without” and (iv) “all” conditions.

Gait speed [cm/s], stride length [cm], step length [cm] of the affected side, maximum hip extensor angle [degree], and maximum ankle plantar flexor moment [Nm/kg] of the affected side for the four conditions are presented in Table 3 (7 patients). Results are averages of more than three periods. Differences were observed in the gait speed, stride length, and step length, although these differences were not significant (n.s.). A significant difference in the maximum hip extension angle of the affected side during the stance phase was observed between (i) “without” and (iv) “all” conditions, as shown in Figure 4. We also found a significant difference in the maximum ankle plantar flexor moment of the affected side between (i) “without” and (iv) “all” conditions (Figure 5).

### 3.2. Effect on Dynamic Joint Stiffness and COP Trajectory in a Patient with Severe Sensory Disorder

Moreover, we thoroughly analyzed data for the patient “E” with the SIAS score for Sensory Function Touch/Position = 0, as shown in Table 1. The SIAS score is widely used for the evaluation of residual function in the field of stroke rehabilitation. Moreover, a previous study reported moderate interclass reliability in Sensory Function Touch/Position, with scores ranging from 0.47 to 0.84 [13].

**Figure 6:**
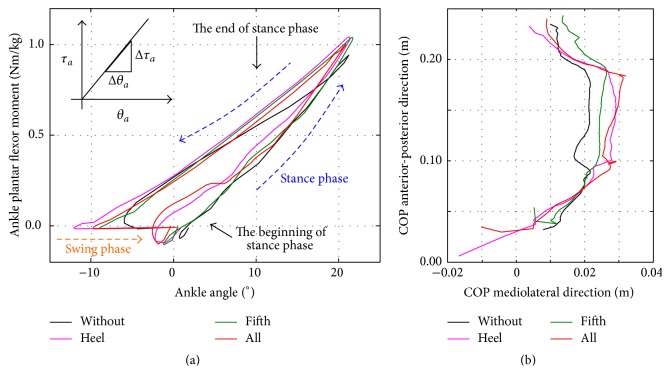
(a) Effects on dynamic joint stiffness of the ankle joint on the affected side: dynamic joint stiffness decreases in the beginning of the stance phase in (ii) “heel” and (iv) “all” conditions, whereas stiffness increases in the late stance phase in (ii) “heel,” (iii) “fifth,” and (iv) “all” conditions, which are similar to that of walking in normal subjects [9]. (b) Effects on COP trajectory on the affected side. The posterior position of COP in the conditions (ii) and (iv) moves backward at the beginning of the stance phase, whereas the anterior position in the conditions (iii) and (iv) moves forward at the end of the stance phase.

Figure 6(a) shows the dynamic joint stiffness of the patient “E” during walking in the four conditions (i)–(iv). The results indicated that dynamic joint stiffness decreases in the beginning of the stance phase in (ii) “heel” and (iv) “all” conditions, whereas stiffness increases in the late stance phase in (ii) “heel,” (iii) “fifth,” and (iv) “all” conditions. These profiles are similar to those of normal subjects [9].  Figure 6(b) represents the effects on the range of COP trajectory on the affected side. These results indicated that the posterior position of COP in (ii) and (iv) moves backward at the beginning of the stance phase, whereas the anterior position in (iii) and (iv) moves forward at the end of the stance phase.

## 4. Discussion

### 4.1. Mechanism Underlying Gait Modification

Past studies in humans and animals have shown that cutaneous receptors in the foot play an essential role in the control of gait [2, 3, 20–29] and posture [30, 31]. In stroke patients with hemiparesis, the range of COP trajectories during walking is narrowed on the affected foot owing to the change in gait [8]. In our prosthesis, we used auditory feedback from the heel and/or fifth metatarsal sensors in conditions (ii), (iii), and (iv). The effects of the prosthesis are summarized as follows: the auditory signal from the heel sensor allowed patients to apply on their heel, and the auditory signal from the fifth metatarsal sensor allowed patients to generate effective pushing off the ground at the end of the stance phase. The first effect was due to the decreasing dynamic joint stiffness at the beginning of the stance phase, whereas the second effect was due to the increasing stiffness at the end of the stance phase, as shown in Figure 6(a). For the first effect, as in the forefoot ice condition [5], COP at the beginning of the stance phase tended to move backward in conditions (ii) and (iv), as shown in Figure 6(b). This would have resulted in the effective knee extension because knee rotation center is located anterior to COP, which, in turn, would have resulted in an increase in the stiffness at the end of stance phase owing to the activation of the gastrocnemius muscle. The two effects contributed to the significant difference in the maximum ankle plantar flexor moment between trials with and without the auditory feedback, resulting in the enhancement of the maximum hip extensor angle of the affected side during the stance phase.

The effects of reduced plantar sensation using an ice immersion technique [5–7] suggested that plantar sensation plays a crucial role in the modification of gait. Reduced plantar sensation resulted in modification of the pressure distribution, with significant reduction of the peak pressure on the heel and toe because of a more cautious touchdown to and push-off from the ground [6]. Furthermore, the hip extension angle at the end of the stance phase is significantly reduced because of similar cautiousness [5]. These results of the reduced plantar sensation corroborate the results of this study, where auditory feedback that enhanced the plantar sensory feedback also enhanced the maximum hip extensor angles of the affected side.

### 4.2. Usability Compared with Previous Approaches

Recently, proposed systems or devices for walking rehabilitation have been mainly classified into three categories: training systems for gait recovery; assistive systems that actively compensate human motor functions; and assistive devices that passively support human motor abilities.* Lokomat* (Hocoma AG, Switzerland) [32],* Autoambulator* (HealthSouth, UK) [33], and* Haptic Walker* (Fraunhofer IPK, Germany) [34], in the first category, are mounted-systems that mostly consist of a weight supporting belt, treadmill, and walking assistive device. A representative device in the second category is* HAL* (Hybrid Assistive Limb) [35] (CYBERDYNE Inc., Japan), which can support and improve human motor function with a robotic system.* Gait Solution* (Pacific Supply Co., Ltd., Japan) [36, 37] and* ACSIVE* (Nambu Co., Ltd., Japan) [37] represent the third category of these devices. However, few past reports have confirmed the effective short- and long-term effects of such devices. As with noninvasive rehabilitation approaches, for example, transcranial magnetic stimulation (TMS) or transcranial direct current stimulation (tDCS), our prosthesis also offers an unconventional, noninvasive approach for walking rehabilitation. The wireless communication system enables expansion of the range of applications to various rehabilitation environments. In the future, we would expect that such smart, wireless, portable, and inexpensive systems may enable widespread use for daily rehabilitation in patients' homes.

### 4.3. Limitations and Future Plans

There are several limitations to the present study. First, the mechanism for gait modification is still not well understood. We focused on the kinematic and kinetic effects of our prosthesis on patient gait, but we did not consider the effects on muscle activation patterns, which are coordinated by the central nervous system (CNS) using sensory information. Recently, muscle synergy patterns, which consist of motor modules as groups of muscles activated at the same time, have been considered as a biomechanical marker associated with pathological conditions [38, 39]. Therefore, it is important to evaluate gait plasticity through the intervention with the proposed prosthesis on the basis of muscle synergy analysis. The relationship between neurological mechanism and biomechanics seems to be of general interest and will also be studied in further investigations.

We found a large variation in standard deviation in Figure 4. The variation was considered to result from the variation in residual function among stroke patients, for example, the difference in Sensory Function Touch/Position. Nonetheless, we found significant differences in maximum hip extension angle and plantar flexor moment in stance between the control and all condition, using ANOVAs followed by Dunnett post hoc tests. This indicates the short-term effect of our prosthesis on walking in stroke patients. Furthermore, we confirmed that the effect of the prosthesis was much larger in patient “E” who has a severe sensory disorder, that is, SIAS score for Sensory Function Touch/Position = 0, as shown in Figures 6(a) and 6(b).

We did not investigate differences in walking performance arising from the location of the sounding source. Kitagawa et al. [40] suggested that spatial modulation of audiotactile interactions might occur for complex auditory stimuli originating from the region close to the back of the head. Thus, we would especially need to consider the location of the auditory feedback source. Furthermore, the coupling or conflict between sensory modalities, for example, visual, vestibular, and somatosensory information, which would significantly affect gait patterns, should also be discussed.

All subjects in the present study were able to walk without any assistance. Our findings cannot be generalized to patients with hemiparesis who use canes and/or orthoses.

In the present investigation, the short-term effect of the prosthesis on walking in stroke patients was successfully confirmed. Rehabilitation is a long-term improvement of dysfunction through the short-term effects of daily interventions. Thus, the long-term effect of our prosthesis will be studied in future. A recent report [41] about a rhythmic motor learning task with visual and auditory signals indicated that the visual feedback (FB) group became dependent on the FB for their performance after the practice, whereas the auditory FB group performed equally well with or without FB after practice. This finding suggests that our auditory feedback prosthesis might eventually allow patients to be less reliant on auditory FB for walking performance after the rehabilitation.

Most studies have reported the effect of impaired plantar sensation on gait plasticity due to aging [42] or diseases, such as diabetes mellitus [43–47] or congenital insensitivity to pain with anhidrosis (CIPA) [48, 49]. Decreased tactile sensation with aging impaired sensory function of limbs and led to falls in the elderly [42]. In patients with diabetic neuropathy, the control of gait and posture is significantly influenced [43]. The reduction in the peak of vertical and horizontal ground reaction force has been reported in patients with diabetic neuropathy [44–46]. Boulton et al. [47] reported that loss of toe function resulted in load shifting from toe to metatarsal head in patients with early diabetic neuropathy. Zhang et al. [49] reported that younger patients with CIPA walked faster, with a longer stride and higher heel contact angular velocity, than controls, owing to sensory impairments. Moreover, modifications of gait and posture have been reported in ischemic blocking [50, 51]. Overall, plantar sensations influence posture and gait in patients with sensory impairments and ischemic block as well as ice immersion [5–7].

Our plan is to apply further rehabilitation efforts in the patients with the following conditions: CIPA [48], Parkinson's disease (PD), and diabetic neuropathy [43–47]. Moreover, we plan to apply the system to experiments involving a prosthetic leg in healthy subjects in a pilot study. Finally, we will verify the long-term effects of the proposed rehabilitation system, as discussed above, leading to brain plasticity of the body representation [52].

## 5. Conclusions

The present investigation showed the short-term effect of the proposed prosthesis on walking in stroke patients with hemiparesis. Significant differences were found in the maximum hip extensor angle and maximum ankle plantar flexor moment with and without auditory feedback from plantar sensations.

## Figures and Tables

**Table 1:** Characteristics of subjects with hemiparesis.

Subject	Gender	Age (years)	Side of hemiparesis (L/R)	Time since stroke (months)	Brunnstrom stage (LE)	SIAS sensory function touch (LE)	SIAS sensory function position (LE)
A	M	60	R	2	V	2	3
B	M	35	L	11	V	2	3
C	M	57	R	23	IV	2	2
D	M	42	L	3	IV	2	3
E	M	65	R	1	VI	0	0
F	M	65	R	95	IV	3	3
G	F	62	L	71	V	2	3

**Table 2:** Segment and placement of markers on the body.

Upper body	
Torso	Spinous process of the 7th cervical vertebrae, spinous process of the 10th thoracic vertebrae, jugular notch where the clavicles meet the sternum, xiphoid process of the sternum, the position in the middle of the right scapula
Upper arm	Both acromions and both lateral epicondyles of elbow
Fore arm	Both lateral epicondyles of elbow, both styloid processes of the ulna, and both styloid processes of the radius

Lower body	
Pelvis	Both anterior superior iliac spines and both posterior superior iliac spines
Upper leg	Both greater trochanters, both lateral epicondyles of knee, and both medial epicondyles of knee
Lower leg	Both lateral epicondyles of knee, both medial epicondyles of knee, both lateral malleoluses, and both medial malleoluses
Foot	Both the first metatarsal heads, both the second metatarsal heads, both fifth metatarsal heads, and both calcaneouses

**Table 3:** The mean and standard deviation of temporal spatial, kinetic, and kinematic data on affected side in the four conditions.

	Without	Heel		Fifth		All	
	Mean (S.D.)	Mean (S.D.)	*p* value	Mean (S.D.)	*p* value	Mean (S.D.)	*p* value
Gait speed [cm/s]	61 (14)	63 (13)	n. s.	64 (13)	n. s.	66 (13)	0.109
Stride length [cm]	85 (12)	89 (11)	n. s.	88 (10)	n. s.	91 (10)	0.066
Step length [cm]	43 (6)	46 (5)	n. s.	45 (5)	n. s.	46 (5)	0.073
Maximum hip extensor angle in stance [degree]	−**2.0 (4.7)**	−1.2 (4.3)	n. s.	−1.1 (4.7)	n. s.	−**0.5 (4.4)**	**0.016**
Maximum ankle plantar flexor moment [Nm/kg]	**0.81 (0.19)**	0.83 (0.22)	n. s.	0.86 (0.22)	0.090	**0.87 (0.20)**	**0.031**
